# Prevalence of inclusion body disease and associated comorbidity in captive collections of boid and pythonid snakes in Belgium

**DOI:** 10.1371/journal.pone.0229667

**Published:** 2020-03-02

**Authors:** Jules Simard, Rachel E. Marschang, Christoph Leineweber, Tom Hellebuyck

**Affiliations:** 1 Division of Poultry, Department of Pathology, Bacteriology and Avian Diseases, Exotic Companion Animals, Wildlife and Experimental Animals, Ghent University, Merelbeke, Belgium; 2 Laboklin GmbH & Co. KG, Bad Kissingen, Germany; CEA, FRANCE

## Abstract

Inclusion body disease (IBD) is caused by reptarenaviruses and constitutes one of the most notorious viral diseases in snakes. Although central nervous system disease and various other clinical signs have been attributed to IBD in boid and pythonid snakes, studies that unambiguously reveal the clinical course of natural IBD and reptarenavirus infection are scarce. In the present study, the prevalence of IBD and reptarenaviruses in captive snake collections and the correlation of IBD and reptarenavirus infection with the clinical status of the sampled snakes were investigated. In three IBD positive collections, long-term follow-up during a three- to seven-year period was performed. A total of 292 snakes (178 boas and 114 pythons) from 40 collections in Belgium were sampled. In each snake, blood and buffy coat smears were evaluated for the presence of IBD inclusion bodies (IB) and whole blood was tested for reptarenavirus RNA by RT-PCR. Of all tested snakes, 16.5% (48/292) were positive for IBD of which all were boa constrictors (34.0%; 48/141) and 17.1% (50/292) were reptarenavirus RT-PCR positive. The presence of IB could not be demonstrated in any of the tested pythons, while 5.3% (6/114) were reptarenavirus positive. In contrast to pythons, the presence of IB in peripheral blood cells in boa constrictors is strongly correlated with reptarenavirus detection by RT-PCR (*P*<0.0001). Although boa constrictors often show persistent subclinical infection, long-term follow-up indicated that a considerable number (22.2%; 6/27) of IBD/reptarenavirus positive boas eventually develop IBD associated comorbidities.

## Introduction

Inclusion body disease (IBD) remains one of the most notorious viral diseases with a global distribution in captive boid and pythonid snakes [1] and is characterized by the presence of eosinophilic or amphophilic intracytoplasmic inclusion bodies (IB) in neurons and glial cells from the central nervous system (CNS), epithelial cells from various organs, smooth muscle tissue, lymphoid cells in esophageal tonsils and peripheral blood cells [2–5].

Although the first study describing clinical signs in IBD positive (IBD^+^) snakes was published in the early 1990s [6], the exact etiology of IBD remained enigmatic for almost two decades. While retroviruses were initially proposed as candidate etiological agents of IBD [6–9], it was not until recently that a causal relationship with novel divergent arenaviruses could be demonstrated [4,5,10–14]. These negative-sense RNA viruses were classified as members of the newly-formed genus *Reptarenavirus*, in the family *Arenaviridae* [15]. Based on a recent in vivo experimental infection study, Koch’s postulates were fulfilled for a *Golden reptarenavirus* (the type species of the genus, previously known as Golden Gate virus, GGV) as a causative agent of IBD [5].

Inclusion body disease has been associated with the development of immunosuppression [7,16] and a variety of clinical signs, such as anorexia, regurgitation, stomatitis, pneumonia, lymphoproliferative disorders and CNS disease, have classically been associated with natural reptarenavirus infection and IBD in snakes [1,2,6,7,17–20]. It remains unclear, however, what primary clinical signs or comorbidities are truly associated with natural reptarenavirus infection and it seems to become more and more obvious that the disease may remain subclinical or show a slowly progressive course in infected snakes [1,5]. The influence of reptarenaviruses on the adaptive immune system of infected snakes has been studied and is likely to play an important role in the disease progression and the development of comorbidities [21,22]. The prevalence of reptarenavirus infection and IBD and the percentage of these asymptomatically infected snakes that eventually will develop clinical signs as well as their role in the disease epidemiology, however, needs to be further elucidated [1,5].

The objectives of the present study were to determine the prevalence of IBD and reptarenavirus infection in captive snake collections and to assess if the probability of the development of comorbidities is related to IBD and reptarenavirus infection. In addition, long-term follow-up of three IBD^+^ snake collections was performed and the agreement of IB detection in stained blood and peripheral white blood cell (PWBC) smears as well as the agreement between IB detection and the results of reptarenavirus RNA RT-PCR testing were evaluated.

## Materials and methods

### Ethics statement

Blood and tissue samples were collected through convenience sampling during entry control testing, routine health assessments or the diagnostic work-up of snakes presented with clinical signs at a veterinary teaching hospital. Blood and tissue samples were subjected to different tests with the owners’ consent. The owners consented both to euthanasia and postmortem sample collection in diseased snakes. No ethical permissions were required for the diagnosis-motivated blood samplings, nor the euthanasia and diagnosis-motivated necropsies of suspected IBD/reptarenavirus infected and diseased snakes (both routine veterinary purposes).

### Animals and sample collection

During a three-year period, whole blood samples were collected from 292 captive boid (Boidae) and pythonid (Pythonidae) snakes from 40 snake collections in Belgium (Table 1). Collections were categorized as closed or open based on the presence or absence of entry control and providing a quarantine period for newly acquired snakes. In three of these collections long-term follow-up of the clinical and IBD status was performed during a three-year period in one collection (collection A) and a seven-year period in two collections (collections B and C), respectively and sampling was performed annually. Collection A was an open and mixed collection that included 85 boid snakes (35 boa constrictors/*Boa constrictor*, 45 reticulated pythons/*Malayopython reticulatus*, two green anacondas/*Eunectes murinus* and three blood pythons/*Python curtus*) of which 10 boa constrictors and 10 reticulated pythons were repeatedly sampled. Collections B and C were closed collections, exclusively consisting of eight and nine boa constrictors, respectively. Whole blood was obtained via cardiocentesis or ventral tail venipuncture and transferred to K3E EDTA tubes (Microvette® 500 μL, Sarstedt) in all sampled snakes.

**Table 1:** Results of inclusion body disease (IBD) and reptarenavirus infection testing in captive boid and pythonid snakes based on the detection of inclusion bodies in hematoxylin and eosin stained blood smears and the detection of reptarenavirus via RT-PCR in blood samples.

	IBD^+^ RT-PCR^-^	IBD^+^ RT-PCR^+^	IBD^-^ RT-PCR^+^	Subtotal	IBD^-^ RT-PCR^-^	Total Sampled Snakes
**Boidae**						
*Acrantophis dumerili*	0	0	0	**0**	9	**9**
*A*. *madagascariensis*	0	0	0	**0**	3	**3**
*Boa constrictor*	6	42	2	**50**	91	**141**
*Calabaria reinhardtii*	0	0	0	**0**	3	**3**
*Candoia aspera*	0	0	0	**0**	1	**1**
*Corallus caninus*	0	0	0	**0**	7	**7**
*C*. *hortelanus*	0	0	0	**0**	1	**1**
*Epicrates cenchria*	0	0	0	**0**	7	**7**
*Eunectes murinus*	0	0	0	**0**	1	**1**
*Sanzinia madagascariensis*	0	0	0	**0**	5	**5**
**Subtotal Boidae**	**6**	**42**	**2**	**50**	**128**	**178**
** *Relative (%)***	*3*.*4*	*23*.*6*	*1*.*1*	*28*.*1*	*71*.*9*	*100*
**Pythonidae**						
*Bothrochilus albertisii*	0	0	0	**0**	4	**4**
*Malayopython reticulatus*	0	0	0	**0**	25	**25**
*Morelia spilota*	0	0	1	**1**	3	**4**
*M*. *viridis*	0	0	0	**0**	7	**7**
*Python bivittatus*	0	0	1	**1**	3	**4**
*P*. *breitensteini*	0	0	1	**1**	3	**4**
*P*. *brongersmai*	0	0	0	**0**	4	**4**
*P*. *curtus*	0	0	1	**1**	4	**5**
*P*. *molurus*	0	0	0	**0**	8	**8**
*P*. *regius*	0	0	1	**1**	42	**43**
*P*. *sebae*	0	0	0	**0**	1	**1**
*Simalia amethistina*	0	0	0	**0**	3	**3**
*S*. *clastolepis*	0	0	1	**1**	1	**2**
**Subtotal Pythonidae**	0	0	6	**6**	108	**114**
*** Relative (%)***	*0*	*0*	*5*.*3*	*5*.*3*	*94*.*7*	*100*
**Total Sampled Snakes**	**6**	**42**	**8**	**56**	**236**	**292**
***Relative (%)***	*2*.*1*	*14*.*4*	*2*.*7*	*19*.*2*	*80*.*8*	*100*

IBD^+/-^: inclusion bodies detected/not detected in hematoxylin and eosin stained whole blood or peripheral white blood cell smears. RT-PCR^+/-^: RT-PCR positive/negative for reptarenavirus. RT-PCR, reverse transcriptase polymerase chain reaction.

### Animal’s clinical status

General physical examination and assessment of the captive management was performed by certified veterinarians (JS, TH) in all sampled snakes. In addition, oropharyngeal and cloacal swabs were collected from all snakes for parasitological examination. Each snake was checked for the presence of ectoparasites. In snakes that showed clinical signs, additional examinations, such as medical imaging, microbiological testing and histopathological examination were performed according to the observed disorder. Snakes were classified as clinically healthy (Ss^-^) or diseased (Ss^+^) based on the absence or presence of clinical signs, respectively. Clinically diseased snakes were further categorized based on the presence or absence of CNS disease (opisthotonus, head tilt, incoordination, tremors, paralyses and delayed righting reflex).

### Blood sample processing

Immediately following blood collection, blood smears were prepared for each sampled snake on a microscopic glass slide (Menzel-Gläser Superfrost®, Thermo Scientific) using standard ‘wedge’ techniques, air dried for 24 hours and H&E stained using a previously published protocol [12]. Next, peripheral white blood cell (PWBC) smears were prepared as previously described by Chang et al. [1] In addition, a K3E EDTA whole blood sample was stored at -21°C until RT-PCR analysis.

### Classification of IBD positive and IBD negative snakes

Snakes were classified as IBD positive (IBD^+^) or IBD negative (IBD^-^) based on the presence or absence of characteristic IB in H&E stained blood smears and PWBC smears using light microscopy with 1000x magnification. Smears were categorized as IBD^-^ if no IB could be detected following the inspection of at least 30 microscopic fields. As soon as a single blood cell with a characteristic IBD inclusion body was detected, the sample was categorized as IBD^+^.

### Reptarenavirus RNA detection by RT-PCR

The studied snakes were classified as reptarenavirus positive (RT-PCR^+^) or reptarenavirus negative (RT-PCR^-^) based on the detection of reptarenavirus RNA via RT-PCR testing. RNA was prepared from 200 μL of the thawed EDTA blood samples using a commercial kit (MagNA Pure 96 DNA and viral NA small volume kit, Roche) according to the manufacturer’s instructions. PCRs for the detection of reptarenaviruses were performed as a conventional PCR using reagents from the RealTime ready RNA Virus Master kit (Roche, Mannheim, Germany) as described previously with a mix of three forward primers (MDS-435: Arena-for1: 5'-TAT ACA ACC AAC GCC CTG TT -3', Arena-for2: 5'-TAC ACA ACC ACA GCC CTG TT -3', Arena-for3: 5'-TAC ACA ACC ACA GCT CTG TT -3') and two reverse primers (MDS-436: Arena-rev1: 5'-AAC ACA TTG GGC CCT TCA C -3', Arena-rev2: 5'-AGC ACA TTG GGC CTT TTA C -3') [10,23]. Specific amplicons were 140 bp long.

### Statistical data analysis

Snakes were categorized as IBD^+^ if IB were detected in H&E stained whole blood and PWBC smears and were considered reptarenavirus infected if they were RT-PCR^+^. The overall prevalence of IBD and reptarenavirus infection was calculated by dividing the number of IBD^+^ and RT-PCR^+^ snakes by the total number of individual snakes included in the study group, respectively. The association between IBD/reptarenavirus infection and clinical signs, age, sex and collection composition were investigated using Fisher’s exact test (clinical signs and collection composition) and chi-square test (age and sex). The agreements between IB detection via whole blood and PWBC H&E stained smears and the agreement between IB detection and the presence of reptarenavirus RNA were assessed by calculating Cohen’s Kappa agreement and using Fisher’s exact test. Kappa values 0, <0.4, 0.4–0.75, >0.75, and 1 were considered as no agreement, poor agreement, good agreement, very good agreement, and perfect agreement, respectively. Differences at *P*≤0.05 were considered statistically significant. Statistical data analysis was performed using commercially available software (GraphPad Prism 5, GraphPad Software).

## Results

### Study group

Samples were obtained from 292 snakes, comprising 114 pythons and 178 boas, belonging to 40 collections (Table 1). The average number of snakes per sampled collection and the average percentage of sampled snakes per collection are depicted in Table 2. For each sampled snake, the age and gender were recorded (Tables 3 & 4). Nine collections were considered as closed collections (9/40) and 31 as open collections (31/40). In addition, collections were categorized as exclusively comprising boas (13/40) or pythons (10/40) or as mixed collection (17/40). Snake blood mite (*Ophionyssus natricis*) infestation was observed in 40.0% (16/40) collections at the time of sampling.

**Table 2:** Number of sampled collections according to the collection size and the average percentage of snakes sampled per collection size category.

Number of snakes per collection	No° of collections	Average % snakes sampled
3–5	14	100%
6–10	10	95%
11–20	4	70%
21–40	8	45%
41–60	2	30%
61 or more	2	20%
**Total**	**40**	

**Table 3:** Relationship between inclusion body disease and reptarenavirus infection and sex in boas (Boidae) and pythons (Pythonidae).

		Male	Female	Unknown	Subtotal	Total
**IBD**^**+**^ **and/or RT-PCR**^**+**^	Boas	24	21	5	**50**	**56**
	Pythons	2	4	0	**6**	
**IBD**^**-**^ **and RT-PCR**^**-**^	Boas	62	63	3	**128**	**236**
	Pythons	51	40	17	**108**	
**Total**		**139**	**128**	**25**	**292**	

Positive male snakes: 18.7% (26/139); Positive female snakes: 19.5% (25/128); Sex unknown positive snakes: 20.0% (5/25). IBD^+/-^: inclusion bodies detected/not detected in hematoxylin and eosin stained whole blood or peripheral white blood cell smears. RT-PCR^+/-^: RT-PCR positive/negative for reptarenavirus. RT-PCR, reverse transcriptase polymerase chain reaction.

**Table 4:** Relationship between inclusion body disease and reptarenavirus infection and age in boas (Boidae) and pythons (Pythonidae).

		Juvenile	Semi-adult	Adult	Subtotal	Total
**IBD**^**+**^ **and/or RT-PCR**^**+**^	Boas	5	16	29	**50**	**56**
	Pythons	2	3	1	**6**	
**IBD**^**-**^ **and RT-PCR**^**-**^	Boas	29	37	62	**128**	**236**
	Pythons	13	46	49	**108**	
**Total**		**49**	**102**	**141**	**292**	

Juvenile: 2 months to 1 year old, 14.3% (7/49) positive snakes; Semi-adult: 1 year to 5 years old, 18.6% (19/102) positive snakes; Adult: 5 years or older, 21.3% (30/141) positive snakes. IBD^+/-^: inclusion bodies detected/not detected in H&E stained whole blood or peripheral white blood cell smears. RT-PCR^+/-^: RT-PCR positive/negative for reptarenavirus. RT-PCR, reverse transcriptase polymerase chain reaction.

### Detection of inclusion bodies and reptarenavirus RNA

**Fig 1:**
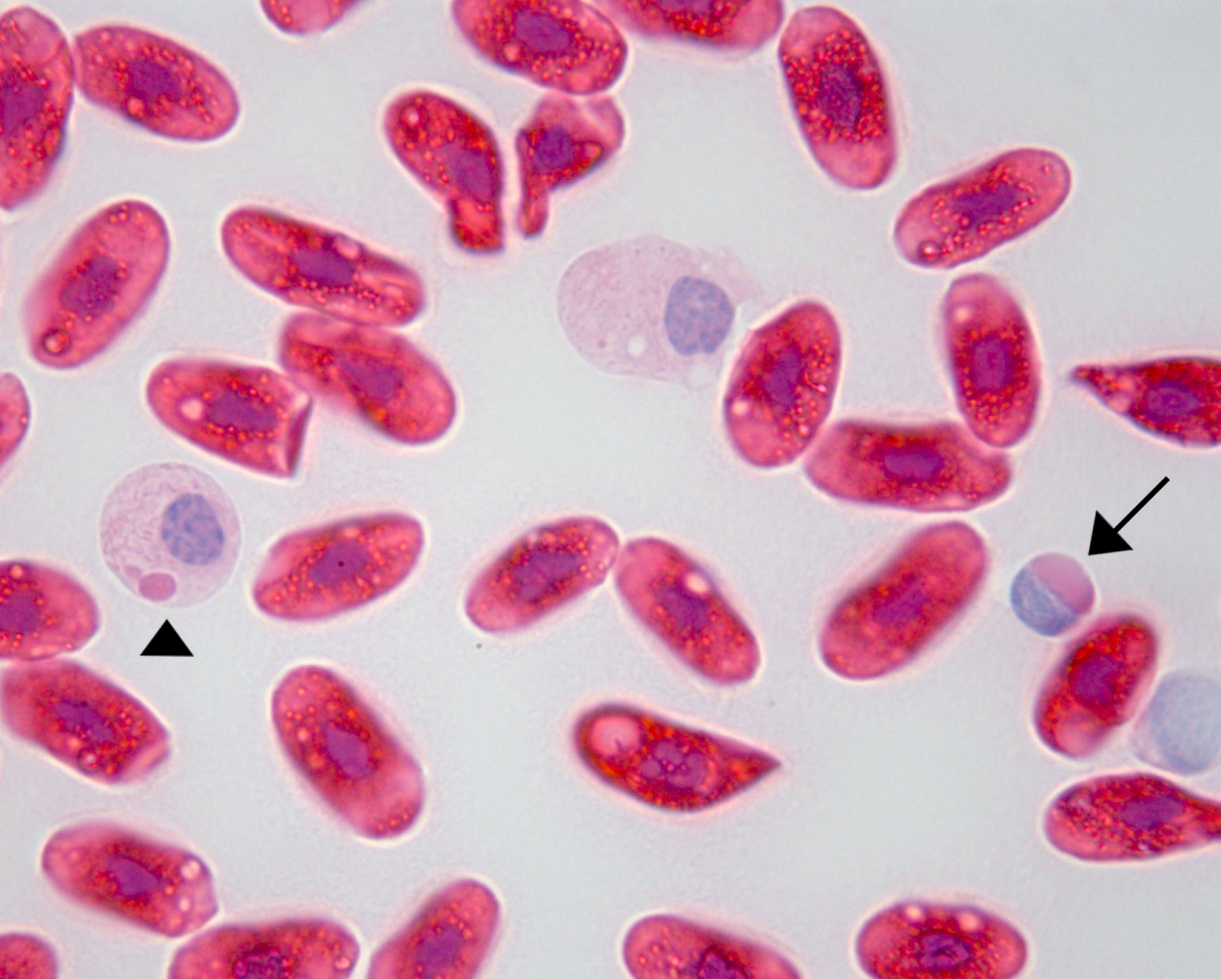
Inclusion body disease in a boa constrictor (*Boa constrictor*). Eosinophilic intracytoplasmic inclusion body in a lymphocyte (arrow) in a hematoxylin and eosin stained blood smear from a reptarenavirus infected boa constrictor (*Boa constrictor*). Occasionally, small eosinophilic intracytoplasmic inclusions were observed in heterophils (arrowhead) (1000x).

Inclusion bodies were exclusively found in boa constrictors (Table 1, Fig 1). The prevalence of IBD^+^ snakes in the present study was 16.5% (48/292), including 27.0% (48/178) of the sampled boas. Among boa constrictors, 34.0% (48/141) were IBD^+^. Reptarenavirus RNA was detected in 17.1% (50/292) of the sampled snakes, including 44 boa constrictors and six pythons. In six IBD^+^ boa constrictors, RT-PCR yielded negative results, while two IBD^-^ boa constrictors were RT-PCR^+^ in the present study. Sanger sequencing of the PCP products of the two IBD^-^ boa constrictors showed 100% identity of approximately 60 bp of the products with the corresponding sequence of University of Giessen virus (GenBank MH503954.1). The overall prevalence of IBD and/or reptarenavirus infected snakes in the present study was 19.2% (56/292).

Among boa constrictors, a very good agreement was found between H&E stained whole blood smears and PBWC smears for IB detection (Kappa agreement = 0.98; *P*<0.0001; Table 5) as well as between IB detection in H&E stained smears and reptarenavirus RNA detection via RT-PCR (Kappa agreement = 0.89; *P*<0.0001; Table 6).

**Table 5:** The agreement between hematoxylin and eosin stained whole blood and peripheral white blood cells smears (PWBC) for the detection of inclusion bodies in boa constrictors (*Boa constrictor*).

	Whole blood^+^	Whole blood^-^	Total
**PWBC**^**+**^	47	1	48
**PWBC**^**-**^	0	91	91
**Total**	47	92	**139**

Observed agreement: (47+91)/139 = 0.99

Kappa statistic = 0.98 (*P*<0.0001)

**Table 6:** The agreement between inclusion body detection in hematoxylin and eosin stained blood and peripheral white blood cells smears and the detection of reptarenavirus RNA by reverse transcriptase polymerase chain reaction (RT-PCR) in blood samples in boa constrictors (*Boa constrictor*).

	IBD^+^	IBD^-^	Total
**RT-PCR**^**+**^	42	2	44
**RT-PCR**^**-**^	6	91	97
**Total**	48	93	**141**

Observed agreement: (42+91)/141 = 0.94

Kappa statistic = 0.89 (*P*<0.0001)

IBD^+/-^: inclusion bodies detected/not detected in hematoxylin and eosin stained whole blood or peripheral white blood cell smears. RT-PCR^+/-^: RT-PCR positive/negative for reptarenavirus.

Among the 50 IBD^+^ boa constrictors, 48.0% (24/50) were male, 42.0% (21/50) were female while the sex was unknown in 10.0% (5/50; Table 3) and 10.0% (5/50) were juveniles, 32.0% (16/50) were semi-adults and 58.0% (29/50) were adults (Table 4). Among the six RT-PCR^+^ pythons, two were male and four were female, of which two were juveniles (one Burmese python/*Python bivittatus* and one ball python/*Python regius*), three were semi-adults (one carpet python/*Morelia spilota*, one blood python/*P*. *curtus*) and one Southern Moluccan python/*Simalia clastolepis*) and one was an adult (Borneo python/*Python breitensteini*). Inclusion body disease was not significantly associated with sex (*P* = 0.9796) or age (*P* = 0.5551). Although a higher prevalence of IBD^+^ boa constrictors was observed in open and mixed collections in comparison to closed collections and collections exclusively consisting of boas, the difference was not statistically significant (*P* = 0.5751 and *P* = 0.5052, respectively).

### Occurrence of clinical signs

Among the 292 snakes included in this study, 249 did not show clinical signs at the time of sampling and 25 showed anorexia or regurgitation that could be unambiguously associated with parasitic infection and/or inadequate husbandry conditions. The latter snakes were excluded from the Ss^+^ group, provided that elimination of the clinical signs was seen following antiparasitic treatment and/or optimization of husbandry. The Ss^+^ group of snakes included 15 boas and three pythons (Table 7). Of the Ss^+^ group, 12 snakes were IBD^+^ and exclusively consisted of boa constrictors of which two boas were IBD^+^/RT-PCR^-^. The latter two snakes were not included in the statistical analysis. Seven boa constrictors and three pythons showed signs of CNS disease. While six out of seven of the latter boas were IBD^+^/RT-PCR^+^, the three pythons were IBD^-^/RT-PCR^-^. The presence of clinical signs was significantly higher in IBD^+^/RT-PCR^+^ snakes in comparison to IBD^-^/RT-PCR^-^ snakes (*P*<0.0001).

**Table 7:** Association between the presence of inclusion bodies disease and/or reptarenavirus infection with clinical signs in boas (Boidae) and pythons (Pythonidae).

		IBD^+^ RT-PCR^-^		IBD^+^ RT-PCR^+^		IBD^-^ RT-PCR^+^		Subtotal	IBD^-^ RT-PCR^-^		Subtotal	Total
		B	P	B	P	B	P		B	P		
**Ss**^**+**^	Respiratory disease	1	0	1	0	0	0	**2**	1	0	**1**	**3**
	Neoplasia	0	0	2	0	0	0	**2**	0	0	**2**	**2**
	Vertebral osteomyelitis	1	0	1	0	0	0	**2**	1	0	**1**	**3**
	Central nervous system disease	0	0	6	0	0	0	**6**	1	3	**4**	**10**
**Subtotal**		2	0	10	0	0	0	**12**	3	3	**6**	**18**
**Ss**^**-**^		4	0	32	0	2	6	**44**	125	105	**230**	**230**
**Total**		6	0	42	0	2	6	**56**	128	108	**236**	**292**

IBD^+/-^: inclusion bodies detected/not detected in hematoxylin and eosin stained whole blood or peripheral white blood cell smears. RT-PCR^+/-^: RT-PCR positive/negative for reptarenavirus. Ss^+/-^: clinical symptoms observed/not observed. B, boas (Boidae). P, pythons (Pythonidae). RT-PCR, reverse transcriptase polymerase chain reaction.

### Long-term follow-up

Collection A was an open and mixed collection consisting of 85, 91 and 82 snakes at the time of sampling during the first, second and third year, respectively. Entry control and quarantine were not performed and the collection was heavily infested with snake blood mites (*Ophionyssus natricis*) throughout the entire follow-up period. Repeated sampling was performed in the same 10 boa constrictors and 10 reticulated pythons that were present at each annual sampling time point. In the first year, four boa constrictors were RT-PCR^+^ of which three were IBD^+^. Although remaining RT-PCR^+^, no IB were found in the IBD^-^/RT-PCR^+^ boa during the entire follow-up period. In the second year, one additional boa constrictor tested IBD^+^/RT-PCR^+^. In the third year, test results were identical to those of the second year. All ten pythons remained IBD^-^/RT-PCR^-^ throughout the entire follow-up period. Initially, none of the tested snakes showed clinical signs but in the third year, two IBD^+^/RT-PCR^+^ boa constrictors as well as three IBD^-^/RT-PCR^-^ reticulated pythons showed CNS disease signs. All of these latter snakes were euthanized and brain and liver tissue were collected. Histopathological evaluation revealed the presence of IB in hepatocytes of the boas. Although no IB were found in tissues from the pythons, non-suppurative meningoencephalitis was diagnosed in all pythons. While liver tissue was RT-PCR^+^ in all boas, brain tissue obtained from the boas and all tissues from the pythons were RT-PCR^-^.

Collections B and C were closed collections, consisting of eight and nine boa constrictors, respectively. In both collections the boas were housed individually, but males were temporarily housed together with females during the breeding period. Snake blood mites were not observed throughout the entire follow-up period. In collection B, four out of eight boa constrictors tested IBD^+^ in the first year, but only two tested RT-PCR^+^. Identical results were obtained during seven consecutive years. One IBD^+^/RT-PCR^+^ boa was euthanized because of progressive vertebral osteomyelitis in the 6^th^ year, but no clinical signs were noticed in the other boas during the entire follow-up period. During the sixth year, a IBD^+^/RT-PCR^+^ female produced nine healthy neonates after mating with an IBD^+^/RT-PCR^-^ male. The offspring tested IBD^-^/RT-PCR^-^ at the age of six, nine, and 12 months. In collection C, four out of nine boa constrictors tested IBD^+^/RT-PCR^+^. Test results were identical during the entire follow-up period. A colonic lymphoma was detected in one IBD^+^/RT-PCR^+^ boa during the fourth year and another IBD^+^/RT-PCR^+^ boa developed an odontogenic fibromyxoma in the fifth year of the follow-up period. In the latter boa, IB and reptarenavirus were detected in blood as well as neoplastic and liver tissue as previously described by Hellebuyck et al. [19] In another IBD^+^/RT-PCR^+^ boa, recurrent respiratory disease responsive to broad-spectrum antimicrobial treatment was noted from the fourth until the last year of the follow-up period. In collection C, a clutch of seven neonates from a IBD^-^/RT-PCR^-^ female and a IBD^+^/RT-PCR^+^ male tested IBD^-^ and RT-PCR^-^ at the age of eleven months.

## Discussion

The overall prevalence of IBD and/or reptarenavirus infection in the present study was 19.2% with a remarkably high prevalence of IBD in boa constrictors (34.0%). Although, a proportionally larger number of boid species other than boa constrictors and pythonids tested reptarenavirus positive in previous screening studies [1,21,24,25], the number of positive snakes that were detected in these studies largely complies to our results. It should be noted, however, that these studies focused on a smaller number of snakes belonging to a single (zoological) collection [21,24,25] or a more limited number of snake collections [1].

In the present study, clinical signs were seen in 25.0% (12/48) of the IBD^+^ snakes, exclusively consisting of boa constrictors, and included bacterial vertebral osteomyelitis, recurrent respiratory disease, neoplastic disorders and CNS disease. Based on our results, these clinical signs may be considered as comorbidities that are significantly associated with IBD/reptarenavirus infection in snakes (P<0.0001). Although immunocompromised snakes may be more susceptible to reptarenavirus infection, the development of comorbidities as observed in this study may also be facilitated by the immunosuppression resulting from arenavirus infection as previously described in other animals with arenavirus infection [26] and recently in reptarenavirus infected boa constrictors [21]. As previously reported [1,24,25], it should be noted that many boa constrictors showed subclinical infections at the moment of sampling and although long-term follow-up was based on a sampling of a limited number of collections, our findings indicate that it may take several years before infection becomes clinical in IBD^+^ or reptarenavirus infected snakes.

A recent study confirmed that pythons rapidly develop CNS disease following experimental inoculation with a reptarenavirus [5]. While no IBD inclusions were found in H&E stained blood smears and PWBC smears and tissue sections, non-suppurative meningoencephalitis was observed in histologic sections from the three pythons from collection A that showed CNS disease indicating viral infection, but an exact etiology could not be demonstrated. Although it remains possible that some reptarenaviruses were not detected by the RT-PCR used in this study, various other infectious but also non-infectious causes may be associated with CNS disease and associated histopathological findings [1,13,27,28,29] as observed in the three pythons from collection A that showed CNS disease. The relatively low number of reptarenavirus infected pythons in our study and the absence of clinical signs in these pythons is a noteworthy finding, especially taking into account the considerable number of sampled mixed collections that included IBD^+^/RT-PCR^+^ boa constrictor and mostly lacked preventive measures against the introduction and transmission of reptarenaviruses. Moreover, many of these collections were heavily infested with snake blood mites which are considered potential vectors of reptarenaviruses [2,16,30]. Importantly, it should be noted that in the present study, with the exception of the euthanized snakes included in the long-term follow-up of collection A, testing for IB and reptarenavirus was limited to blood samples and routinely available diagnostic methods. Accordingly, the true prevalence of IBD and reptarenavirus infection may be underestimated, especially in the sampled pythons, as it is generally accepted that IB and reptarenaviruses may be confined to the CNS in pythons [5]. The use of other diagnostic testing modalities such as immunohistochemical (IHC) staining could have increased the sensitivity and specificity towards the detection of reptarenavirus nucleoprotein in blood smears and samples collected for histopathological examination [1,12] and IBD detection in early infection stages [4]. Although recent studies of the snake adaptive immune response to reptarenavirus infection in boa constrictors demonstrated inconsistency in anti-reptarenavirus antibody formation in infected snakes [22] and an apparent negative correlation between IBD and anti-reptarenavirus antibodies [21], serological testing could be attempted to detect (non-viraemic) reptarenavirus infection [21]. The use of oral and cloacal swabs as well as sampling of the esophageal tonsils for RT-PCR testing has been described and could increase the sensitivity of reptarenavirus infection testing in combination with other testing modalities [24,25]. More research is needed to assess the sole or adjuvant diagnostic value of these diagnostic methods in the antemortem diagnosis of reptarenavirus infection in boid and pythonid snakes.

In contrast to what has been described by Hyndman et al. [24], serial testing performed during the long-term follow-up of three collections did not reveal considerable changes in the number of IBD^+^ or RT-PCR^+^ snakes that were detected. It should be mentioned that boa constrictors were overrepresented in the collections that were subjected to long-term follow-up, while Hyndman et al. [24] mainly performed testing in pythons. Based on the methods used in this study, vertical transmission of reptarenaviruses as described by Keller et al. [4] and Aqrawi et al. [23] could not be demonstrated through testing of juvenile boa constrictors from collections B and C. In the study of Keller et al. [4], IB as well as reptarenavirus RNA could be detected at an age of eight months in blood samples obtained from juvenile boas that were vertically infected. For this reason, the likelihood of demonstrating vertical transmission in the offspring from an IBD^+^/RT-PCR^-^ father and IBD^+^/RT-PCR^+^ mother of collection B that was tested at an age of nine and 12 months and the offspring from an IBD^+^/RT-PCR^+^ father and IBD^-^/RT-PCR^-^ mother of collection C tested at an age of 11 months was deemed to be considerably high based on the performed IB and RT-PCR testing. It should be taken into account that vertical transmission might not have occurred in the tested offspring of collection C if the mother was not infected with reptarenavirus during co-habitation and mating with the IBD^+^/RT-PCR^+^ father. As previously discussed, however, reptarenaviruses can escape RT-PCR detection and this could have contributed to the obtained negative results in the tested offspring from both collections [1]. Besides serial testing of the offspring from collection B during a prolonged time period, postmortem sample collection (including brain tissue) for IB detection based on H&E and/or IHC staining as well as reptarenavirus RT-PCR might have provided more certainty towards the occurrence of vertical transmission in the tested juvenile boas [4,23].

A very good agreement was found for IB detection in blood smears compared to detection in PWBC as well as between the presence of IB and the detection of reptarenavirus in blood samples from boa constrictors. No IB were detected in any of the sampled pythons in this study, supporting the findings of former studies indicating that reptarenavirus infected pythons do not routinely develop IB in circulating blood cells [1,5,24,25]. In addition, IB were not detected in blood and PWBC smears of two RT-PCR^+^ boas, suggesting that similar to pythons, the absence of IB does not rule out reptarenavirus infection in boa constrictors, especially in the earliest stage of reptarenavirus infection and in vertically infected neonatal snakes [4,5,25]. The fact that several IBD^+^ boa constrictors consistently tested RT-PCR^-^ (collection B) is remarkable. Although it cannot be fully excluded, it is very unlikely that the detection of IB in IBD^+^/RT-PCR^-^ boas was unrelated to reptarenavirus infection as these IB had the typical appearance of IBD inclusions in H&E stained smears [3,12,13,24,25,31] identical to those found in IBD^+^/RT-PCR^+^ boas in the present study. As previously reported, however, reptarenaviruses are highly genetically diverse [3,4,14] and although the RT-PCR that was applied in the present study has a high sensitivity comparable to a similar PCR that allowed the detection of a wide range of reptarenaviruses [3] and has been used to detect reptarenaviruses in a large variety of boid and pythonid snakes and vipers [23], it is possible that some RT-PCR^-^ results might have been false negatives as some reptarenaviruses might escape detection by RT-PCR due to mutations in the primer binding regions [3,4,13,14,24].

## Conclusion

The results of this large-scale study demonstrate that IBD and reptarenaviruses are highly prevalent in captive boa constrictors and that both boas as well as pythons can act as asymptomatic carriers of reptarenaviruses. The presence of IBD^+^/RT-PCR^+^ boa constrictors does not seem to contribute to increased IBD associated morbidity at the level of a collection, but a considerable number of chronically IBD^+^ boa constrictors seem to eventually develop IBD/reptarenavirus associated comorbidities. Our findings suggest that evaluation of H&E stained blood and PWBC smears combined with RT-PCR testing of blood samples have an excellent predictive value towards the diagnosis of IBD/reptarenavirus infection in semi-adult and adult boa constrictors. In pythons and in early reptarenavirus infection stages, however, results of antemortem diagnosis based on these methods should be cautiously interpreted as IB and reptarenavirus RNA do not seem to be readily detected in blood samples.
